# Importation-Driven Measles Resurgence and the Fragility of Elimination, Japan, 2024–2026

**DOI:** 10.3201/eid3211.261187

**Published:** 2026-11

**Authors:** Koichiro Yuji, Wakako Yuji, Eisuke Adachi

**Affiliations:** The Institute of Medical Science, The University of Tokyo, Tokyo, Japan (K. Yuji, E. Adachi); Kitasato Institute Hospital, Tokyo (K. Yuji, W. Yuji, E. Adachi)

**Keywords:** measles, viruses, importation, measles elimination, mass gatherings, vaccination coverage, respiratory infections, modified measles, surveillance, Japan

## Abstract

As measles elimination unravels across the Americas, Europe, and the Western Pacific, Japan is experiencing a resurgence of measles; the World Health Organization had verified elimination of measles in Japan in 2015. By epidemiologic week 20 of 2026, a total of 498 cases had been reported, exceeding every full-year total since 2020. Median case-patient age has fallen to 26 years, shifting the burden from a generation who received single-dose measles vaccinations to younger cohorts caught between successive vaccination-policy transitions. Among case-patients with a documented 2-dose vaccination history, more than half experienced modified measles, an attenuated, easily missed illness that delays diagnosis and isolation. As mass gatherings concentrate global travel, Japan has become a sentinel for the fragility of measles elimination, including in countries that consider elimination status secure. Surveillance shows importation-driven resurgence; the shift in genotype profile from D8 to B3 predominance back to D8 enabled tracking of source countries.

In November 2025, the World Health Organization (WHO) Region of the Americas lost its regional elimination status after endemic measles transmission was reestablished in Canada, which had maintained elimination since 1998 (*1*). Transmission has since accelerated: by epidemiologic week 28 of 2026, the region had reported 47,459 confirmed cases and 44 deaths, >3 times the 2025 total and the highest count since 2004 (*2*). The Pan American Health Organization (PAHO) Regional Verification Commission, which reviews all countries in the region annually, will consider the 2026 reports, including that of the United States, in November 2026 (*1*,*3*). By September 10, 2026, the United States had recorded 3,294 confirmed cases, its largest annual total since elimination was declared in 2000; of the cases, 95% were outbreak-associated and 95% occurred in unvaccinated persons or those of unknown status (*4*,*5*). The percentage internationally imported has fallen to ≈10%, signaling a shift toward sustained domestic transmission (*3*). 

The erosion of measles elimination status is not confined to the Americas. The WHO European Region recorded 127,350 measles cases in 2024, double the 2023 total and the highest in >25 years (*6*); on the basis of that resurgence, WHO announced in January 2026 that 6 countries, including the United Kingdom, had lost their elimination status. The age structure for those cases is instructive: among cases reported in Europe during January–June 2026, half (51%) were among those >15 years of age, and 15% of those with known vaccination status had received 1 dose (*7*).

Japan’s own region is a cautionary backdrop. Measles in the WHO Western Pacific Region rose 743% during 2022–2024; Mongolia and Cambodia, the first middle-income countries there to eliminate measles, later lost that status to importation-related outbreaks (*8*,*9*). Japan, where elimination was verified in 2015 (*10*), had recorded more cases by mid-2026 than in any full year since 2020. We argue that Japan is a sentinel for an elimination framework under growing strain, with lessons extending well beyond the Western Pacific to every country whose elimination status now rests on fragile foundations.

## A Rapid, Geographically Dispersed Resurgence

This report draws on Japan’s mandatory national surveillance data. By epidemiologic week 20 of 2026, a total of 498 cases had been notified through Japan’s National Epidemiologic Surveillance of Infectious Diseases, already exceeding every full-year total since 2020 (Figures 1, 2). The lowest years, 2020–2022, had 6–10 cases annually, coinciding with stringent COVID-19 control measures and the near-cessation of international travel, so they do not represent a normal baseline. Measured instead against the prepandemic period, 2025 and 2026 still mark a clear departure from the postelimination norm (*12*). Weekly counts in 2026 were 28–39 cases across weeks 11–14; they peaked at 62–71 in weeks 15–17 and fell to 17–23 in weeks 18–20, a decline the national report attributes partly to reduced holiday-period reporting (*13*) (Figure 2).

**Figure 1:**
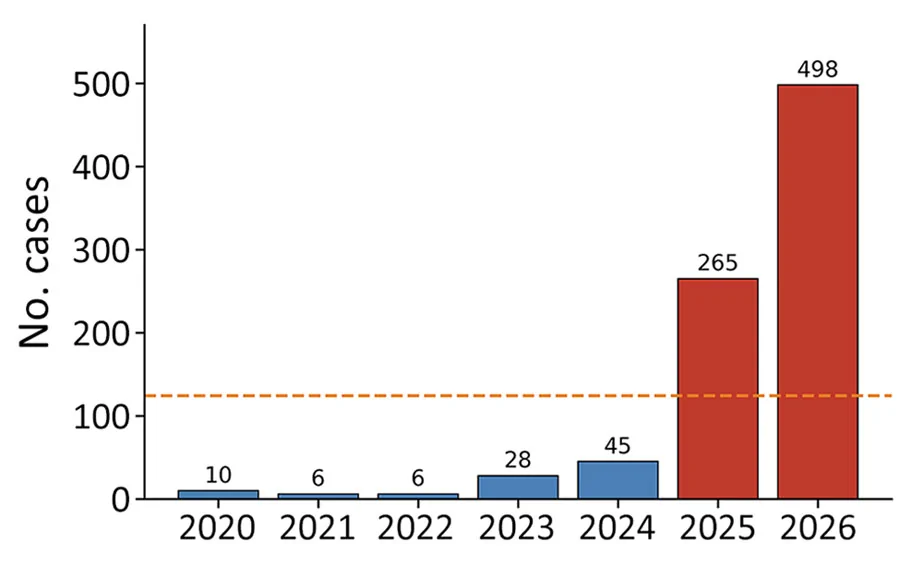
Annual reported cases of measles, Japan, 2020–2026. Red bars denote provisional totals not yet finalized for 2025 and all 2026 data for epidemiologic weeks 1–20. Blue bars represent finalized annual counts for 2020–2024. Dashed line marks an incidence of 1 confirmed case/1 million population/year (≈124 cases/year for Japan), a World Health Organization surveillance performance indicator for monitoring progress toward and maintenance of elimination rather than a criterion for verification, which rests on multiple lines of evidence (*11*).

**Figure 2:**
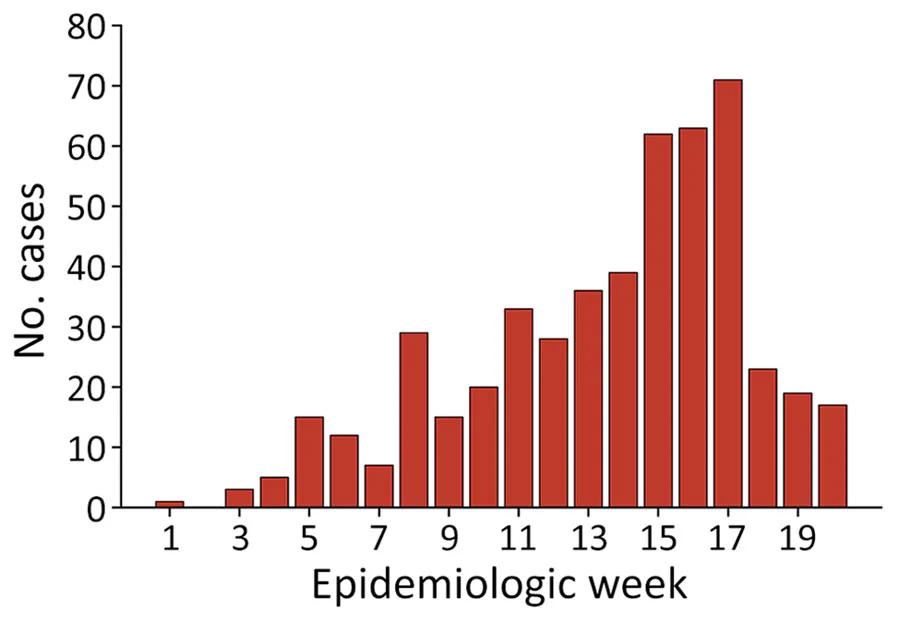
Weekly cases of measles by week of diagnosis, Japan, 2026. Provisional data for epidemiologic weeks 1–20, through May 20, 2026, are shown. Counts rose from week 11, peaked in week 17, and fell in weeks 18–20; the decline might reflect reduced reporting over an extended holiday period. Because reports accrue over subsequent weeks, counts for the most recent weeks are provisional and will rise.

We derived detailed epidemiologic, genotype, and clinical data from the national featured report for the first 20 weeks of 2026 (498 cases; data as of May 20, 2026), the most granular dataset available at the time of writing. Cases were identified in 27 of 47 prefectures, indicating multiple concurrent transmission chains rather than 1 localized outbreak. The amplifying potential of even 1 importation is illustrated by an imported case reported in March 2024 that seeded a superspreading event, including secondary transmission to fellow passengers on the same flight, with the highest transmissibility concentrated in the first generation of spread before self-isolation and public-health awareness curtailed further transmission (*14*). That episode shows how 1 importation can ignite a rapidly amplifying cluster despite nominally high population vaccination, a dynamic that recurs wherever susceptibility is unevenly distributed.

## Importation Sources and Genotype Succession

The resurgence of measles in Japan is importation-driven; genotype profiles are linked closely enough to the source countries to function as a molecular record of travel patterns. In 2023, importations from Indonesia and India predominated; genotype D8 accounted for 88% of typed cases (*15*). In 2025, Vietnam became the leading source of imported infection, accounting for ≈83% of cases with an overseas-acquired source; genotype B3 surged to 87% (*16*). By 2026, Indonesia had reemerged as the leading overseas source. Genotype information was available for 341 (69%) of the 498 cases reported through week 20; among those, genotype D8, one of the 2 genotypes (with B3) responsible for most measles transmission globally in the study period, accounted for 61% and B3 for 39% of cases, reversing the ratio seen earlier in the resurgence (*13*). The geography of those cases fits an importation-anchored process. Five prefectures accounted for just over half of all modified measles cases during 2016–2025, yet incidence in neighboring prefectures was uncorrelated in every period examined; cases clustered around metropolitan gateways rather than diffusing across adjacent prefectures. The leading prefectures also moved eastward between resurgence years, from Osaka and Kanagawa in 2019 to Kanagawa and Ibaraki in 2025, over the same period in which the dominant genotype and source countries changed (*16*). That shifting genotype profile—D8 predominance in 2023, a shift to B3 predominance by 2025, and a reversal to D8 predominance in 2026—mirrors the shifting epidemiology of the Asia-Pacific source countries on which Japan’s importation risk depends and underscores that domestic case counts are inseparable from the regional control situation.

Interpreting those data against the formal definition of elimination requires caution. WHO criteria defined reestablishment of endemic transmission as uninterrupted circulation of the same measles virus genotype and lineage for >12 months; elimination is verified only after 36 months of interrupted transmission (*11*). Our analysis is limited to genotype-level frequencies and cannot resolve named strains or lineages. The standard 450-nt window of the nucleoprotein gene is not sufficiently discriminating to separate endemic from imported virus within a genotype; whole-genome sequencing, particularly of the noncoding region between the matrix and fusion genes, carries the phylogenetic information needed to distinguish sustained domestic chains from repeated importations (*17*). Because 31% of cases are ungenotyped, the apparent absence of a single sustained lineage is provisional. We emphasize this fact because genotype-frequency data alone can neither establish nor exclude loss of elimination.

## Adult Susceptibility and the Legacy of Vaccination-Policy Transitions

The measles resurgence is concentrated in young adults, although not the same adults as in 2019. Among the 498 cases reported in Japan through week 20 of 2026, median patient age was 26 (range 0–65) years (*13*). Age-specific incidence locates that shift (Figure 3): it peaked at 30–34 years in 2019 but at 20–24 years in 2026, two 5-year bands lower; combined incidence at 30–39 years fell to about 80% of its 2019 value (*12*,*13*,*18*). That shift traces back to Japan’s national immunization program.

**Figure 3:**
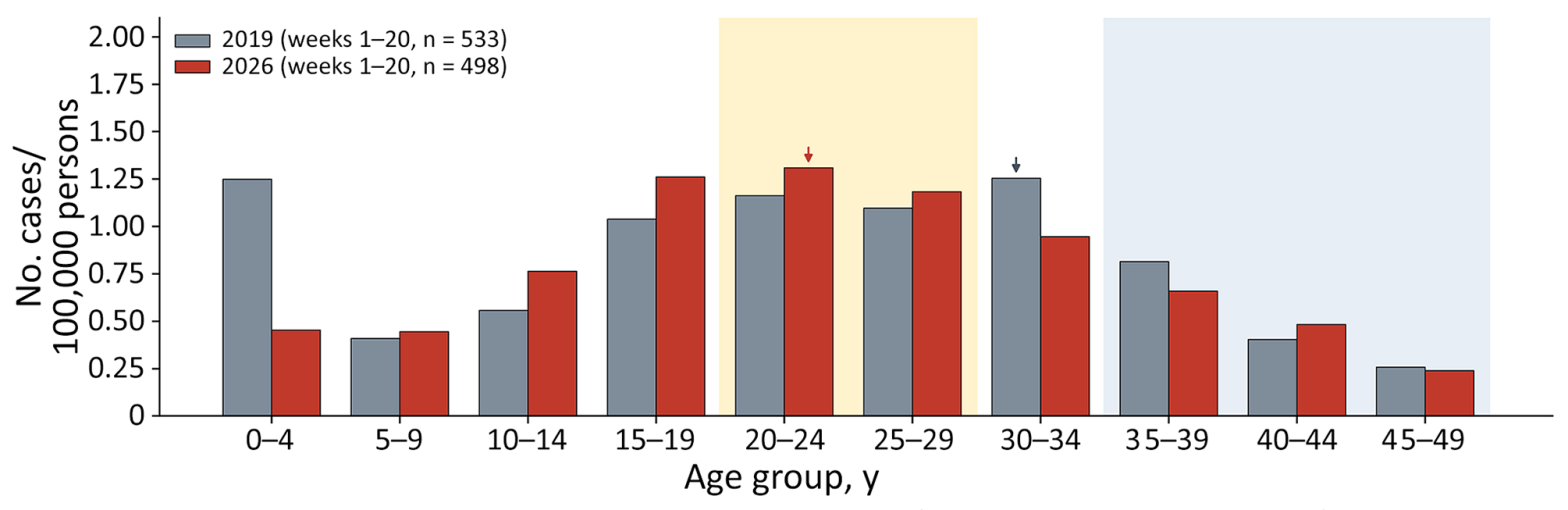
Measles cases by age group reported in epidemiologic weeks 1–20 of 2019 and 2026, Japan. Age-specific incidence in weeks 1–20 of 2019 (n = 533) and 2026 (n = 498) were calculated from national case counts and population estimates for the corresponding year. Shading indicates 2 cohorts defined by vaccination policy. Yellow shading indicates the age groups of the transitional cohorts, born ≈1995–2005, who received a second dose of measles vaccine, if they did so, through the time-limited 2008–2013 campaign or the opening years of the 2-dose schedule. Light blue shading indicates the single-dose cohort, born ≈1978–1990, who were offered 1 routine dose and were not eligible for the second dose through the campaign. Black arrow indicates peak age group in 2019; red arrow indicates peak age group in 2026. Source: Japan Institute for Health Security Infectious Disease Weekly Report (*13*,*18*,*19*).

Measles vaccine entered Japan’s routine immunization program in October 1978, administered as a single dose to young children (*20**,**21*). The schedule remained single-dose for >25 years. A structural change came in 1994, when an amendment to the Immunization Act ended mandatory vaccination, shifting measles immunization from a compulsory to a recommended basis (*22*). Persons born from 1978 until the routine 2-dose schedule began in 2006 (i.e., for persons 21–48 years of age in 2026) were offered a single routine dose for part of that period on a noncompulsory basis. A single dose confers seroconversion in ≈93% of recipients, so most of that population is protected, and susceptible persons are a small fraction of it.

The 2-dose measles–rubella schedule was introduced in 2006, prompted by WHO Western Pacific Region’s 2005 resolution to eliminate measles; the first dose was given to children 12–24 months of age and the second before elementary school entry (*11*,*22*). A nationwide resurgence in 2007–2008 among teenagers with a single dose exposed the gap that the new schedule could not close for those past school-entry age (*23*,*24*). Japan responded with a 5-year catch-up supplementary immunization activity during April 2008–March 2013, offering a second dose to adolescents in the first year of junior high school and the final year of high school (*23*,*24*). By reaching adolescents of specific ages at that time, the campaign created a set of transitional cohorts: persons born during 1995–2005 received their first dose under the single-dose regime and their second, if at all, through a time-limited campaign or the opening years of the 2-dose schedule.

Read against that history, the 2 resurgences implicate different cohorts. The adults infected in 2019 were born predominantly in the late 1980s; they were vaccinated under the single-dose schedule and already too old for the 2008–2013 catch-up. That single-dose generation was, in the 2019 resurgence, the decisive susceptible population. By 2026, both the median case-patient age and the peak of age-specific incidence fell within the transitional cohorts, born in 1995–2005, whose second dose came through a time-limited campaign or the opening years of the 2-dose schedule, if at all (Figure 3). The immunity gap has not closed so much as moved from a generation offered 1 dose to younger cohorts whose 2 doses were given inconsistently with few opportunities for natural boosting in a country where measles no longer circulates; that distinction is supported by evidence that, in a setting in which measles was eliminated, measles antibodies wane after vaccination but not after infection (*25*). We attached no point estimate to the susceptible population because coverage data cannot distinguish between primary-vaccine failure, waning immunity, and unrecorded catch-up doses (*16*).

Compounding the weakness in the historical structure, contemporary routine coverage has begun to slip. First-dose measles–rubella coverage was 95.4% in fiscal year 2022 (Japan’s fiscal year runs April–March); in fiscal year 2024, first-dose coverage was 92.7% and second-dose coverage 91.0%, both below the 95% level associated with interrupting transmission (*15*,*26*). A cohort-structured susceptibility and an eroding contemporary coverage were therefore acting in combination to increase risk for infection and spread. We observed a comparable combination underlying the contemporaneous loss of regional measles elimination in the Americas, where PAHO has assessed the regional public-health risk as very high because of declining coverage and increases in susceptible populations (*2*); the parallel underscores a wider pattern.

## Diagnostic Challenge of Modified Measles in Vaccinated Adults

A clinically consequential feature of measles resurgence is the appearance of infection in vaccinated adults. That observation is not evidence that vaccine failure is becoming more common; in the United States, the breakthrough fraction has remained stable at ≈8%–12% of reported cases, and the 2025 resurgence occurred overwhelmingly among unvaccinated persons or those with no documented vaccination (*5*,*27*). What such settings share is not a rising rate of vaccine failure but pockets of susceptibility within populations whose aggregate coverage appears adequate, differing in how those pockets are defined: by community in the United States, by birth cohort in Japan, and, in Israel’s outbreak of ≈3,200 cases traced to a single importation, by childhood nonvaccination (median age 5.6 years, 83% unvaccinated) (*28*). Among the 159 cases in 2026 with 2 documented doses (158 of them in patients >6 years of age), 89 (56%) experienced modified measles, an attenuated illness characterized by a subtle rash, minimal or absent fever, and frequently absent Koplik spots (*13*,*29*); that proportion applies to documented 2-dose recipients only, because vaccination status was unknown for 41% of case-patients >6 years of age. In adults, the manifestation is easily mistaken for a nonspecific viral exanthem or a drug reaction, particularly because clinicians in elimination settings might not consider measles in a vaccinated patient at all. Clinical suspicion is lowest in the population in which such infection occurs.

Characterizing the transmission risk for modified measles accurately is important because both overstatement and understatement carry costs. In a systematic review of postelimination settings, index case-patients with secondary vaccination failure produced few secondary infections among their contacts; secondary attack rates were 0%–6.25%, and the estimated effective reproduction number for onward transmission from such cases was 0.063. Onward transmission from such cases is therefore substantially less efficient than from a case in an unvaccinated person, but it does occur, and clusters have been documented under conditions of close and prolonged contact (*30*). The principal hazard posed by modified measles is therefore diagnostic rather than directly epidemiologic: because its attenuated manifestation delays recognition, isolation, and contact tracing, more transmissible infections could seed in susceptible contacts. In the largest United States series, comprising 4,056 cases during 2001–2022, breakthrough infections accounted for 475 (12%) of cases, were milder, and arose predominantly from secondary rather than primary vaccine failure (*27*). Vaccinated adults will increasingly be included in case series wherever elimination is under pressure.

The practical implications for clinicians are concrete. Physicians evaluating an adult with febrile rash should elicit a vaccination and travel history, keep measles on the differential even in 2-dose recipients, and conduct both IgM serology and reverse transcription PCR, because IgM could be transiently negative early in modified measles. Of importance, the mildness of the index case does not diminish the public health stakes. Each unrecognized case can transmit to those at greatest risk: infants too young to be vaccinated, pregnant persons, and immunocompromised persons. Historically, ≈30% of reported cases have experienced complications; the risk for complications is highest in children <5 years of age and in adults (*31*). Measles also induces immune amnesia, depleting immune memory for years after infection (*32*). A milder index case is not benign.

## Uncertainty of Low-Transmission Estimates for Japan

A caveat specific to the Japan context bears directly on how the reassuringly low transmission estimates for secondary vaccination failure should be read. Two distinct susceptible groups must be separated. The first comprises fully (2-dose) vaccinated adults who nonetheless develop breakthrough infection as modified measles; for those, the low transmission estimates derived from secondary vaccination failure apply, and the dominant hazard is diagnostic delay rather than efficient onward spread. The second, larger group comprises adults whose birth cohorts were never offered a routine 2-dose schedule or who were offered a second dose only through time-limited catch-up campaigns of incomplete uptake; vaccine failure here occurs not in the immunological sense but by a structural absence of a second dose across whole birth cohorts. Conflating the two would understate the transmission potential of the resurgence because the parameters that reassure for the first group do not describe the second.

That distinction also qualifies how the secondary vaccination failure estimates themselves should be generalized. Those estimates derive overwhelmingly from highly 2-dose–vaccinated populations, in which the rare breakthrough case is surrounded by immune contacts, and onward transmission is correspondingly contained. The containment observed in such settings is a property not only of the index case-patient’s attenuated infectiousness but also of the surrounding immune environment. Where that environment is itself partially susceptible, as it is where Japan’s transitional cohorts are concentrated, the same low per-case transmission probability can nonetheless seed longer chains because a larger fraction of contacts are themselves at risk. Low-transmission parameters measured in 2-dose populations may therefore not extrapolate to a setting in which the susceptible fraction is generational. That conclusion does not imply that modified measles is highly transmissible; it cautions against importing reassurance from populations whose immune structure differs from Japan’s. The same caution applies wherever pockets of susceptibility persist within an otherwise highly vaccinated population, whether defined by birth cohort, by community, or, as in much of Europe, where half of cases are now in adolescents and adults, by incomplete 2-dose rollout.

## Mass Gatherings

The timing of this resurgence magnifies its stakes. The 2026 FIFA World Cup, held June 11–July 19 across the United States, Canada, and Mexico, brought millions of travelers into 3 host nations that were simultaneously experiencing active resurgences and maintaining coverage below the 95% threshold (*1*). Measles has a well-documented history of transmission at mass gatherings, where large numbers of persons from areas of differing immunity mix in crowded, often indoor settings. During the 1991 International Special Olympics, measles spread to 16 persons across 7 US states, including spectators exposed by airborne transmission in a domed stadium; 9 additional cases resulted from onward transmission (*33*). The episode demonstrated that 1 infectious person in a large enclosed venue can transmit measles across substantial distances and subsequently across state and national boundaries.

Sporting events involving youth have repeatedly served as foci. At a 2007 international youth sporting event, the index case-patient was a 12-year-old participant from Japan infected with genotype D5 measles; contact tracing across 8 US states identified 7 cases, 71% of whom were in unvaccinated persons (*34*). Given that measles has a basic reproduction number of 12–18, among the highest of any human pathogen (*31*), a single importation into a mass gathering can ignite rapid and geographically dispersed spread before public health authorities are aware that transmission has begun.

The measles risk associated with the 2026 World Cup has been recognized mainly from the perspective of the host region (*35*). Japan’s experience adds a complementary view: that of a participating Asia-Pacific nation that is simultaneously a major source of outbound travelers and a recipient of importation-linked cases. Japan is thus embedded in a bidirectional risk network rather than positioned only as a potential importer or exporter. The 20th Asian Games in Aichi–Nagoya, Japan, during September–October 2026, shortly after the World Cup, leaves the Western Pacific region facing its own overlapping mass-gathering season. Because travel medicine frames measles as a borderless threat in which every nonimmune traveler is a potential carrier and vector (*36*), defending elimination becomes a shared transnational responsibility that no single country can discharge alone.

## Implications for Surveillance and Response

Japan’s measles resurgence points to 4 priorities that generalize to other elimination-era settings. First, catch-up vaccination should follow the gap as it moves toward the cohorts born between the end of the single-dose regime and the maturation of the 2-dose schedule; Japan’s 2008–2013 adolescent program showed that coverage in a defined age band can be raised within a finite period. Second, pretravel vaccination checks should be integrated into routine travel-medicine practice, given that importation drives nearly every domestic chain. Third, second-dose coverage should be restored to >95% in every prefecture. Fourth, surveillance data on vaccination status, genotype, and transmission chains should be completed and standardized; the large percentages of missing data limit the resolution at which transmission can be understood. Because the disease burden concentrates in gateway prefectures rather than spreading contiguously, strengthening awareness and laboratory confirmation in those prefectures is likely to yield more than uniform national measures.

Beyond those immediate measures, the resolution of transmission chains in future outbreaks would benefit from analytic approaches that aggregate surveillance cannot provide. Generation-dependent modeling and transmission-network analysis, which have been applied to previous measles outbreaks in Japan, offer a route to reconstructing who-infected-whom structure and to distinguishing sustained endemic transmission from repeated independent importations; those distinctions are central to the formal assessment of elimination status. Pairing such methods with systematic genomic sequencing would replace genotype-frequency snapshots of the kind reported here with lineage-resolved chains, materially strengthening the evidentiary basis for elimination decisions.

Our analysis relied on aggregate routine surveillance, which may underconfirm modified measles, given its diagnostic difficulty. Vaccination status was unknown for 41% of eligible 2026 cases, genotype data covered 69% of cases, and place of infection was unknown for 18% of cases. Transmission chains could not be resolved from aggregate data, and no phylogenetic analysis was available, so the genomic findings reported here are descriptive genotype frequencies rather than resolved lineages. We calculated incidence (Figure 3) from aggregate case counts and population denominators, not individual vaccination histories; we determined incidence by age but could not establish differential susceptibility by vaccination status, for which serosurveillance would be required. Finally, 2025 and 2026 case counts are provisional and subject to retrospective revision. Those limitations argue for, rather than against, the strengthening of surveillance that we recommend.

## Conclusions

Japan’s measles resurgence is a sentinel signal. A nation with historically high vaccination coverage, robust surveillance, and verified elimination is losing ground, and it is doing so through mechanisms that are not unique to Japan: cohorts left between successive vaccination-policy transitions, eroding contemporary coverage, importation pressure, and underrecognized infection in vaccinated adults. What differs among settings is the structure of susceptibility rather than the fact of exposure; Japan’s gap is cohort-structured and policy-derived, whereas recent transmission in North America has been concentrated in undervaccinated communities. That distinction matters, because it determines whether catch-up should be targeted by birth cohort or by community; the underlying dynamic of accumulated susceptibility meeting sustained importation pressure is shared. As elimination falters across the Americas and Europe and mass gatherings concentrate global travel, the lesson is that elimination is reversible everywhere, including in settings that consider it secure. Yet, as PAHO has emphasized, status that is lost can also be regained through coordinated immunization and surveillance. The reversibility cuts both ways. Defending measles elimination depends as much on clinical vigilance, keeping measles on the differential in vaccinated adults and confirming it by laboratory testing, as on population-level coverage, and it depends on recognizing that in an interconnected world the susceptibility of one country is the concern of all.
